# Country of infection among HIV-infected patients born abroad living in French Guiana

**DOI:** 10.1371/journal.pone.0192564

**Published:** 2018-02-08

**Authors:** Mathieu Nacher, Leila Adriouch, Astrid Van Melle, Marie-Claire Parriault, Antoine Adenis, Pierre Couppié

**Affiliations:** 1 Centre d’Investigation Clinique Antilles Guyane, CIC INSERM1424 ; Centre Hospitalier Andrée Rosemon, Cayenne, French Guiana; 2 Coordination Régionale de la lutte contre le VIH (COREVIH Guyane) Centre Hospitalier Andrée Rosemon, Cayenne, French Guiana; 3 Service de Dermatologie Vénéréologie, Centre hospitalier Andrée Rosemon, Cayenne, French Guiana; Instituto de Salud Carlos III, SPAIN

## Abstract

**Background:**

Over 75% of patients in the HIV cohort in French Guiana are of foreign origin. Our objective was to estimate what proportion of the migrant population of HIV-infected patients in Cayenne had been infected in French Guiana.

**Methods:**

We included patients of known foreign origin who were followed in Cayenne, for whom the year of arrival in French Guiana was known and the initial CD4 count at the time of diagnosis was available. The time between seroconversion and time at diagnosis was estimated using the formula [square root (CD4 at seroconversion)-square root(CD4 at HIV diagnosis)] / slope of CD4 decline.CD4 counts at the time of infection and the slope were computed in an age and ethnicity-dependent variable.

**Results:**

The median estimated time between infection and diagnosis was 4.5 years (IQR = 0.2–9.2). Overall, using a median estimate of CD4 count at the time of infection, it was estimated that 53.2% (95% CI = 48.3–58%) of HIV infected foreign patients had acquired HIV after having arrived in French Guiana. Patients having arrived in French Guiana before and during the 1990s and those receiving their HIV diagnosis before 2010 were more likely to have been infected in French Guiana.

**Conclusions:**

Contrary to widespread belief suggesting that most migrants are already HIV-infected when they arrive in French Guiana, a large proportion of foreign HIV patients seem acquire the virus in French Guiana.There is still much to do in terms of primary prevention and testing among migrants.

## Introduction

French Guiana is the French overseas territory most affected by the HIV/AIDS epidemic. HIV prevalence in pregnant women has exceeded 1% for over 2 decades and AIDS incidence is ten times higher than the national average (21.6 per 100,000 *vs*. 2.1 per 100,000)[1,2]. Although, the situation is often described as a “generalized epidemic” the epidemiology shows that it is mostly concentrated in vulnerable groups [3]. Transmission is mostly heterosexual (86% compared to 39% in mainland France) with the HIV cohort including as many men as women [4]. Multiple heterosexual partnerships and/or concurrent relationships among men combined with a lack of consistent condom use are contributing factors[5]. Prostitution, having tried crack cocaine and being a migrant risk factors in French Guiana [3]. Thus, 29% over the population in French Guiana is of foreign origin and over 75% of patients in the HIV cohort in French Guiana are of foreign nationality, a situation that is unusual in other Latin American countries. A hypothesis is that migrants come from areas of high prevalence and concentrate high risk groups that thus acquire HIV outside of French Guiana; another complementary hypothesis is that migration and poverty constitute a context where sexual risks increase. Indeed, a study conducted in 2012 among migrants in French Guiana showed that over a third of the surveyed persons were frequently engaging in risky sex within French Guiana and were thus at high risk of acquiring HIV. Multiple concurrent sexual partnerships and transactional sex were more frequent that in the rest of the population. About 2/3rds of current sex partners were of similar origin, but2/3rds reported occasional sex partners of another country of origin in French Guiana (Personal communication, Submitted manuscript).

In Europe, the proportion of infections acquired in European countries in non-MSM Africans with HIV was variable and ranged between 2 and 37% [6]. Although methodological differences complicated the comparisons, the studies converged in the conclusion that there is on-going sexual transmission after the arrival in the host country. Methods based on CD4 count decline and date of arrival have been used to estimate the probable country of infection and they have yielded a larger estimated proportion of post migration HIV acquisition than studies based on clinic reports.[7]

In this context, our objective was to determine what proportion of the migrant population of HIV-infected patients followed in Cayenne General Hospital had probably been infected in French Guiana. Acquiring this knowledge ultimately aimed to facilitate advocacy for increased resources for primary prevention and testing among migrants in French Guiana.

## Methods

### Study design

#### Setting and participants

Data on HIV patients in French Guiana has been available since 1989. Clinical, biological and epidemiological data was entered by specific trained research technicians in the DMI2 government software until 2008, and in eNADIS/DATAIDS[8], and now transferred on the DOMEVIH government software since 2017.

#### Inclusion criteria

We included patients of known foreign origin who were followed in Cayenne, for whom the year of arrival in French Guiana was known and the initial CD4 count at the time of diagnosis was available.

We did not include patients from Saint Laurent du Maroni, a border town where it is more complicated to determine when persons actually lived on the territory or from Kourou where the information on date of arrival was not available.

### Statistical methods

#### Likely place of infection

The year of infection was estimated for each patient for whom the year of arrival in French Guiana was available. This estimation was based on the rate of CD4 decline, which depended on each person’s age and ethnicity, between the CD4 count at the time of diagnosis and the estimated CD4 count at the time of HIV infection. CD4 cell count at HIV sero-conversion and square root of CD4 cell decline were obtained using the method described in the UK[7,9] which studied the CD4 decline in a cohort of HIV-infected persons, fitting random effect (slope and intercept) multilevel linear regression models.This allowed to calculate CD4 counts at the time of infection and the slope in an age and ethnicity-dependent variable.[7,9]Most migrants French Guiana are from African American ancestry thus the median CD4 count and interquantile range, and the slopeof CD4 (square root) decline used were those calculated for black populations (median 487(IQR = 377–619)), and 0.2+0.02*age, respectively.[7,9] For persons from northern Brazil, who are often of mixed Amerindian ethnicity we used median CD4 count and interquantile range, and the slopeof CD4 (square root) for other ethnicities (median 538(IQR = 403–701)), 0.55+0.02*age. The time between seroconversion and time at diagnosis was estimated using the formula [square root(CD4 at seroconversion)-square root(CD4 at HIV diagnosis)]/ slope of CD4 decline.[7,9]

#### Statistical analysis

The statistical analysis was performed using Stata 13.0 (Stata Corp LP, College Station, TX, USA).

Descriptive statistics were obtained. A binary variable was generated that was coded 1 for those presumed to have acquired HIV in French Guiana and 0 for those presumed to have acquired HIV outside of French Guiana.

A simple and a multivariate regression model were constructed to determine which simple sociodemographicvariables(age group (15–30, 31–40, 41–50, >50 years), gender, nationality, CDC stage, year of diagnosis (1989–1996, 1997–2002, 2003–2010, 2011–2016) were associated with acquiring HIV in French Guiana. Categorical variables were used as sets of indicator variables. Given the frequency of the outcome prevalence ratios were used instead of odds ratios to avoid distortion.[10]The statistical significance threshold was set at 5%. The Pearson goodness of fit test was used.

#### Ethics and funding

Patients included in the FHDH give written informed consent for the use of their case record data for research and publication of research results. Patient identity is encrypted before sending the data to the Institut National de la RechercheMédicale (INSERM), which centralizes data from Regional Coordinations for the fight against HIV (COREVIH) throughout France. The cohort was approved by the Commission NationaleInformatiqueetLibertés (CNIL) since Nov 27th 1991 and has led to numerous international scientific publications. No specific funding was obtained.

## Results

Overall there were 840 known foreign patients in the outpatient clinic at Cayenne general hospital which represented 81.15% of patients of known nationality (for 191patients nationality was not available in the medical file). Of the 840 foreign patients, 524 came from the Caribbean (62.4%), and 286 came from South America (34% of migrants). Most of the remaining 4% came from Africa. Overall 489 (58.2% of migrants) came from Haiti. S1 and S2 Tables show the overall country of origin of patients in Cayenne General Hospital outpatient clinic and the country of origin of patients with an available date of arrival and initial CD4 count at diagnosis used to compute the duration of infection.

**Fig 1:**
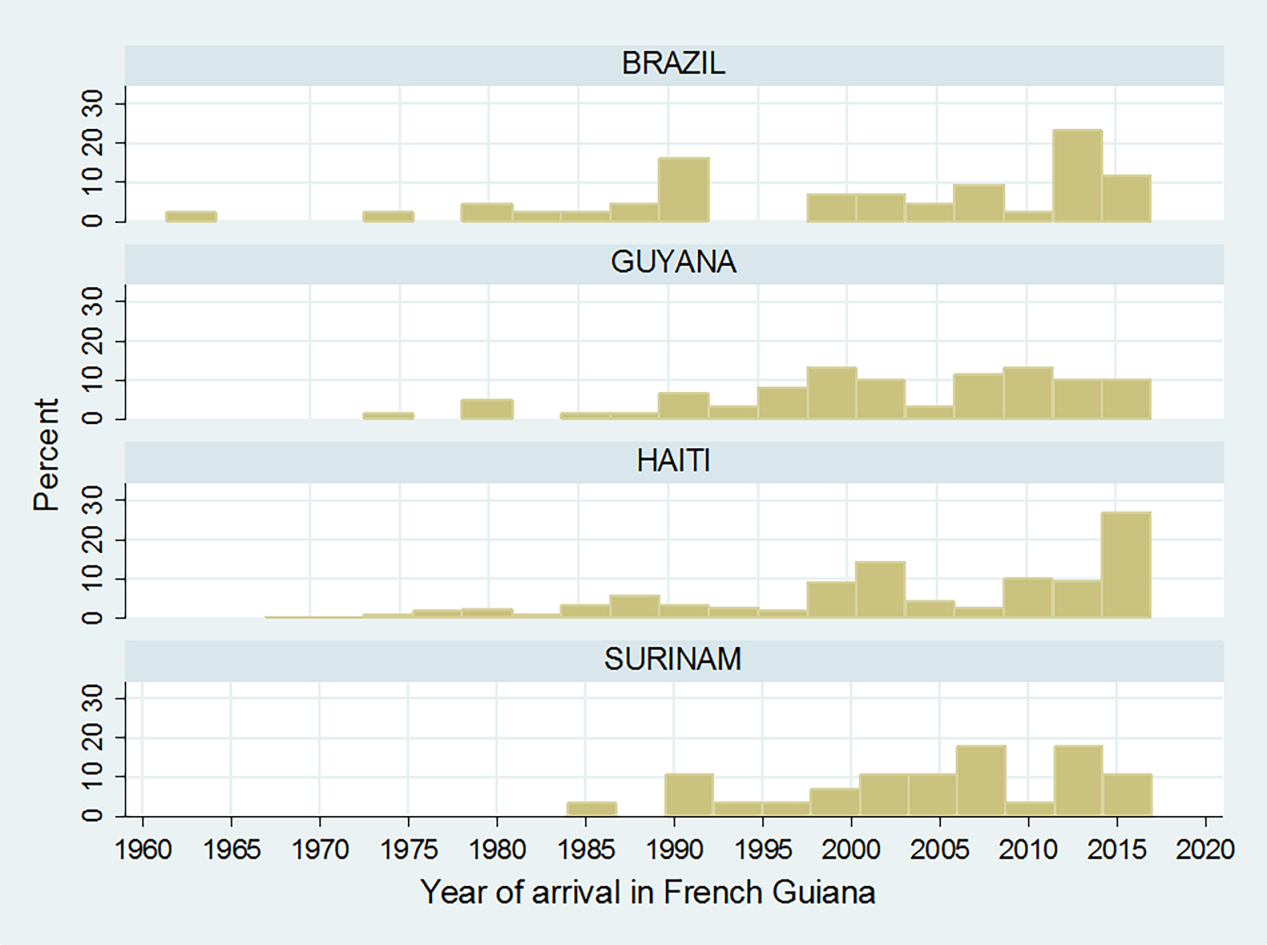
Proportion of patient by year of arrival for the 4 main countries of origin of HIV-positive immigrants in Cayenne outpatients.

Data on arrival year and CD4 count at diagnosis was available for 421 patients. Fig 1 shows the proportion of patient by year of arrival for the 4 main countries of origin of HIV-positive immigrants in Cayenne outpatients. There was a recent massive immigration wave from Haiti in 2014–2016.

The median CD4 count at the time of diagnosis in foreign patients was 292 per mm3 (IQR124-481), with differences between countries: Brazil 245 per mm3 (IQR = 98–384), Guyana 366 per mm3 (IQR = 200–537), Haiti 310 per mm3 (138–491), Surinam 258 per mm3 (107–448), p<0.001 (Kruskal Wallis test).

Estimations of the proportion of foreign patients having acquired HIV in French Guiana.

Overall, depending on the median, 25% or 75% estimates of CD4 count at the time of infection [7] 53.2% of HIV-infected patients (25% 75% estimates 43.7% and 64.4%, respectively) acquired HIV after having arrived in French Guiana.

Table 1 shows different scenarios based on initial CD4 estimates and the resulting 95% confidence intervals.

**Table 1:** Estimates of proportion of HIV infections acquired within French Guiana for different scenarios based on initial CD4 estimates and the resulting 95 confidence intervals.

	Number of infected in French Guiana/number outside of French Guiana	Proportion of HIV infections acquired in French Guiana (95% CI)
**CD4 at seroconversion: median estimate used in computations**	224/197	53.2 (48.3–58)
**CD4 at seroconversion: 1^st^ quartile used in computations**	271/150	64.3 (59.5–69)
**CD4 at seroconversion: 3^rd^ quartile used in computations**	184/237	43.7 (38.9–48.6)

The median estimated time between infection and diagnosis was 4.5 years (IQR = 0.2–9.2) with significant differences between countries of origin (Brazil 5.3 years, Guyana 3.1 years, Haiti 3.8 years, Surinam 5.6 years, P<0.001, Kruskal Wallis test).

Table 2 shows that among the tested predictors of HIV acquisition in French Guiana having arrived in French Guiana for a longer period was more likely to be associated with acquiring HIV in French Guiana whereas recent HIV diagnoses (after 2010) were more likely to be associated with acquiring HIV abroad.

**Table 2:** Predictors of HIV acquisition in French Guiana.

** **	**Crude prevalence ratio (95%CI)**	Adjusted prevalence ratio* **(95%CI)**	**P**
**Country**			
**Brazil**	0.9 (0.6–1.5)	0.8 (0.4–1.4)	0.4
**Haiti**	Ref	Ref	
**Guyana**	0.9 (0.6–1.4)	0.9 (0.6–1.5)	0.8
**Suriname**	0.8 (0.4–1.4)	0.9 (0.5–1.9)	0.9
**Other**	1.8 (0.9–3.5)	1.3 (0.2–10.5)	0.7
** **			
**Arrival decade**			
**<1990s**	3.2 (2.2–4.6)	9.4 (4.8–18.5)	<0.001
**1990–2000**	2.3 (1.6–3.5)	5.5 (3.1–9.9)	<0.001
**2000–2010**	1.4 (0.9–2)	2.3 (1.4–3.9)	0.002
**>2010**	Ref	Ref	
** **			
**Year of HIV diagnosis**			
**<1990**	2 (1.2–3.2)	0.8 (0.1–5.8)	0.8
**1990–2000**	1.4 (1–2.1)	0.6 (0.3–1)	0.05
**2001–2010**	1.2 (0.9–1.7)	0.6 (0.4–0.9)	0.01
**2011–2016**	Ref	Ref	Ref
** **			
**CDC stage (present)**			
**A**	Ref	Ref	
**B**	1.1 (0.7–1.7)	0.9 (0.6–1.5)	0.8
**C**	0.9 (0.7–1.3)	0.7 (0.51.08)	0.1
**Transmission mode**			
**Homo/bisexual**	0.7 (0.3–1.5)	0.7 (0.3–2.1)	0.5
**Heterosexual**	Ref		
**Other**	0.7 (0.3–1.5)	1 (0.4–2.5)	0.9
** **			
**Age category (years)**			
**[15–30]**	Ref	Ref	
**]30–40]**	1 (0.5–1.8)	0.6 (0.3–1.2)	0.17
**]40–50]**	1.2(0.7–2.2)	0.7 (0.3–1.3)	0.2
**>50**	1.7 (0.9–2.9)	0.5 (0.2–1.1)	0.09
**Sex**			
**M**	1 (0.8–1.3)	0.9 (0.7–1.3)	0.6
**F**	Ref		

*Goodness of fit test *P* = 1.

Table 3 shows that for Haitian nationals, for whom there has been a recent migration wave following disasters in Haiti, predictably there was a significant trend of being more likely to be infected in Haiti than in French Guiana.

**Table 3:** Proportion of HIV acquisition in French Guiana for Haitian nationals by time period.

**Period of arrival in French Guiana**	infected in French GuianaN(%)	Infected in Haiti N(%)	Odds*
**1960–1990**	71 (100)	0 (0)	.
**1991–2000**	53 (71.6)	21 (28.4)	2.52
**2001–2010**	52 (41.6)	73 (58.4)	0.71
**2011–2017**	48 (31.8)	103 (68.2)	0.46

*Chi square for linear trend, P<0.001.

## Discussion

The present results suggest that in French Guiana 53% (1^st^ and 3^rd^quartiles64 and 43%) of HIV infections in migrants had been acquired within French Guiana. Increased sexual risks in migrants have been described in several studies worldwide and French Guiana is no exception [11,12]. Isolation, the ensuing decrease of community regulation of sexuality, vulnerability and poverty converge to increase the migrants’ likelihood of engaging in sexual risks. This result is higher than what was observed in mainland France (49% the difference is not statistically significant, P = 0.18)[13] or in the UK (33%, P<0.001)[7]. In contrast with France there was no significant difference between men and women. Age groups, CDC stage, country of origin or by transmission mode were not significantly associated with any difference in the risk of acquiring HIV within French Guiana. The decade of arrival in French Guiana was independently associated with a higher likelihood of having acquired HIV in French Guiana than more recent arrivals.Similarly more recent HIV diagnoses were independently less likely to have been acquired within French Guiana. The fact that year of arrival and year of diagnosis are both associated with the probability of having acquired HIV in French Guiana are consistent with the fact that the longer the persons remain in French Guiana the greater is the probability of acquiring the HIV virus and getting diagnosed and followed in HIV outpatient care. The results suggested that patients from Suriname and Brazil had a longer delay between infection and diagnosis, perhaps reflecting the fact that Maroon populations from Suriname and Brazilian Garimpeiros often live in remote areas where HIV testing is less accessible. Finally, the association of more recent HIV diagnoses with HIV acquisition abroad may reflect recent efforts to improve HIV testing among newly arrived immigrants.

The present study has a number of potential weaknesses. First this modeling exercise is based on medians obtained from multiethnic cohorts in the UK, who may not apply to the exact population structure of French Guiana. However, the initial CD4 and slope estimates included populations from the Americas and computed values for these populations. The year of arrival is not systematically recorded in medical records and thus was not available for half of our cohort. Often for some patients from bordering Brazil or Suriname there is an uncertainty on which side of the border the patient is, and thus no arrival date was noted. In any case there was no significant difference in the median CD4 count at the time of diagnosis between patients with an arrival date and those with a missing arrival date. The second main center for HIV care in French Guiana Saint Laurent du Maroni was not included for 2 main reasons: the lack of information on date of arrival in the medical records, and mostly the fact that numerous patients have pendular migrations from one side of the border to the other or even reside in Suriname and only consult in Saint Laurent for reasons linked to fear of stigma in Suriname, availability of the most recent drugs, or the possibility of getting residence and work permits. It is thus not easy to determine the date of arrival when most patients move of both sides of the border and when some are reticent to disclose that they do not live in French Guiana for fear of not being eligible for health care. This study thus only gives us a picture of the “capital” Cayenne and cannot be inferred to border areas which would deserve a specific study. However, Cayenne hospital follows 2/3 of HIV patients in French Guiana and thus this information has strategic value. Finally, migrants may go back to their home country and thus get infected there while residing in French Guiana, which could lead to misclassification of the place of infection. However, these trips are often short and thus the probability of this happening on a large scale seems low.

Overall, there is still much to do in terms of primary prevention and testing among migrants. Contrary to widespread belief suggesting that most migrants are already HIV-infected when they arrive in French Guiana, a large proportion of foreign HIV patients seem acquire the virus in French Guiana.This is important and timely data for French Guiana given the recent massive wave of immigrants from Haiti following hurricane [14–16].

## Supporting information

S1 Table Country of origin of patients in Cayenne General Hospital outpatient clinic. (DOCX)

S2 Table Country of origin of patients with an available date of arrival and initial CD4 count at diagnosis used to compute the duration of infection. (DOCX)
