# Supplementary material for: Country of infection among HIV-infected patients born abroad living in French Guiana
Source: PLoS One. 2018 Feb 8;13(2):e0192564. doi: 10.1371/journal.pone.0192564 (PMC5805311; doi:10.1371/journal.pone.0192564)
Supplement: S1 Table — (DOCX) [file pone.0192564.s001.docx]

Supplementary table 1. Country of origin of patients in Cayenne General Hospital outpatient clinic

| **Country of Origin** | **Freq.** | **Percent** |
| --- | --- | --- |
| **Algeria** | 1 | 0.12 |
| **Argentina** | 1 | 0.12 |
| **Brazil** | 98 | 11.67 |
| **Cameroon** | 3 | 0.36 |
| **Colombia** | 3 | 0.36 |
| **Ivory coast** | 4 | 0.48 |
| **Dominica** | 3 | 0.36 |
| **Ethipia** | 1 | 0.12 |
| **Gambia** | 1 | 0.12 |
| **Ghana** | 1 | 0.12 |
| **Guinea** | 1 | 0.12 |
| **Guinea-Bissau** | 9 | 1.07 |
| **Guyana** | 135 | 16.07 |
| **Haiti** | 489 | 58.21 |
| **Madagascar** | 1 | 0.12 |
| **Pakistan** | 1 | 0.12 |
| **Peru** | 4 | 0.48 |
| **Portugal** | 2 | 0.24 |
| **dominican Republic** | 20 | 2.38 |
| **Saint Lucia** | 12 | 1.43 |
| **Senegal** | 3 | 0.36 |
| **Suriname** | 45 | 5.36 |
| **Togo** | 2 | 0.24 |
|  |  |  |
| **Total** | 840 | 100.00 |
