# Supplementary material for: Country of infection among HIV-infected patients born abroad living in French Guiana
Source: PLoS One. 2018 Feb 8;13(2):e0192564. doi: 10.1371/journal.pone.0192564 (PMC5805311; doi:10.1371/journal.pone.0192564)
Supplement: S2 Table — (DOCX) [file pone.0192564.s002.docx]

Supplementary table 2. Country of origin of patients with an available date of arrival and initial CD4 count at diagnosis used to compute the duration of infection.

| **Country of origin** | **Freq.** | **Percent** |
| --- | --- | --- |
| **Brazil** | 43 | 10.24 |
| **Guinea** | 1 | 0.24 |
| **Guinea-Bissau** | 1 | 0.24 |
| **Guyana** | 59 | 14.05 |
| **Haiti** | 282 | 0.67 |
| **Peru** | 1 | 0.24 |
| **Portugal** | 1 | 0.24 |
| **Dominican Republic** | 5 | 1.19 |
| **Suriname** | 28 | 6.67 |
|  |  |  |
| **Total** | 420 | 100.00 |
